# Supplementary material for: Epidemiological characteristics of respiratory viruses in children during the COVID-19 epidemic in Chengdu, China
Source: Microbiol Spectr. 2023 Dec 5;12(1):e02614-23. doi: 10.1128/spectrum.02614-23 (PMC10783071; doi:10.1128/spectrum.02614-23)
Supplement: Supplemental Figure S1 — The differences in age and the different viral infections. Flu A = influenza A; Flu B = influenza B; PIV I = parainfluenza virus I; PIV II = parainfluenza virus II; PIV III = parainfluenza virus III; ADV = adenovirus, RSV = respiratory syncytial virus, D = Day, M = Month, Y = Year. [file spectrum.02614-23-s0001.docx]

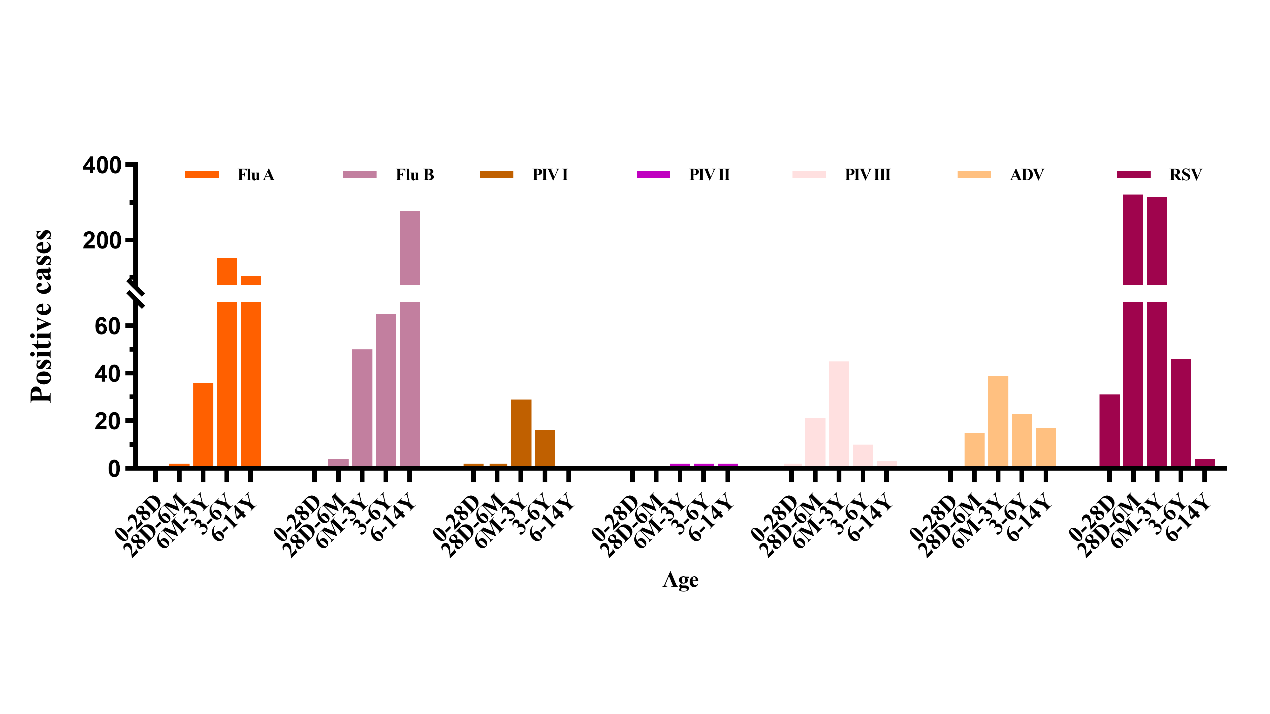


Supplement figure1 the differences in age and the different viral infections. Flu A =influenza A; Flu B= influenza B; PIV I= parainfluenza virus I; PIV II= parainfluenza virus II; PIV III =parainfluenza virus III; ADV = adenovirus, RSV = respiratory syncytial virus, D= Day, M=Month, Y=Year.

Table 1 The sociodemographic variables of the study subjects.

D= Day, M=Month, Y=Year
